# Interventions to prevent preterm birth following fetoscopic laser surgery for twin‐to‐twin transfusion syndrome: systematic review and meta‐analysis

**DOI:** 10.1002/uog.29230

**Published:** 2025-06-05

**Authors:** N. Eltaweel, F. D'Antonio, S. Prasad, H. Mustafa, A. Khalil

**Affiliations:** ^1^ Division of Biomedical Science, Warwick Medical School, University of Warwick University Hospital of Coventry and Warwickshire Coventry UK; ^2^ Center for Fetal Care and High‐Risk Pregnancy University of Chieti Chieti Italy; ^3^ Vascular Biology Research Centre Molecular and Clinical Sciences Research Institute, St George's University of London London UK; ^4^ Department of Obstetrics and Gynecology, Division of Maternal‐Fetal Medicine Indiana University School of Medicine Indianapolis IN USA; ^5^ The Fetal Center at Riley Children's Health Indianapolis IN USA; ^6^ Fetal Medicine Unit, St George's University Hospitals NHS Foundation Trust University of London London UK; ^7^ Twins and Multiples Centre for Research and Clinical Excellence, St George's University Hospital, St George's University of London London UK; ^8^ Fetal Medicine Unit, Liverpool Women's Hospital University of Liverpool Liverpool UK

**Keywords:** cerclage, pessary, preterm birth, prevention, progesterone, twin pregnancy, twin‐to‐twin transfusion syndrome

## Abstract

**Objective:**

To assess the impact of intervention with cervical cerclage, cervical pessary or vaginal progesterone on the risk of preterm birth (PTB) in monochorionic diamniotic (MCDA) twin pregnancies undergoing fetoscopic laser surgery (FLS) for twin‐to‐twin transfusion syndrome (TTTS).

**Methods:**

The MEDLINE, Embase and Cochrane databases were searched from inception to November 2023. The inclusion criteria were studies on MCDA twin pregnancies undergoing FLS for TTTS, comparing those receiving with those not receiving an intervention to prevent PTB, including vaginal progesterone, cervical cerclage and cervical pessary. The primary outcome was gestational age (GA) at birth. The secondary outcomes included the interval between FLS and birth, PTB prior to 34, 32, 28 and 24 weeks' gestation, delivery within 2 and 4 weeks after FLS, preterm prelabor rupture of membranes, chorioamnionitis, double survival, survival of at least one twin, no survival, overall fetal or perinatal loss, and overall fetal or perinatal survival. All outcomes were explored in the overall population of MCDA twin pregnancies undergoing FLS for TTTS according to different cut‐offs of cervical length (CL) for intervention. Random‐effects meta‐analysis was used to directly compare the risk of each outcome. The Grading of Recommendations, Assessment, Development and Evaluation (GRADE) methodology was used to assess the quality of the retrieved evidence.

**Results:**

Ten studies (1159 MCDA pregnancies) were included in the systematic review, of which seven were included in the meta‐analysis. There was no significant difference in mean gestational age at birth in MCDA twin pregnancies undergoing FLS for TTTS in women receiving *vs* not receiving cervical cerclage, with CL < 30, < 25, < 20 or < 15 mm. There was also no significant difference in the mean interval between FLS and delivery, and in the risk of fetal or perinatal loss between women receiving *vs* not receiving cervical cerclage. Similarly, intervention with cervical pessary was not associated with a higher gestational age at birth compared with no intervention. It was not possible to perform any comprehensive pooled data synthesis for women receiving progesterone. In women with CL < 30 mm, intervention with cervical pessary was not associated with a reduced risk of PTB < 32, < 28 or < 24 weeks' gestation, or with delivery within 2 or 4 weeks after FLS or perinatal loss. Finally, in women with CL < 25 mm, cervical pessary did not reduce the risk of PTB < 32 weeks or perinatal loss. On GRADE assessment, the quality of evidence was very low in showing that cervical cerclage and cervical pessary can affect gestational age at birth in MCDA twin pregnancies that underwent FLS for TTTS, irrespective of the degree of cervical shortening.

**Conclusions:**

There is currently no evidence that intervention with cervical cerclage or pessary leads to a greater gestational age at birth or reduces the risk of PTB in MCDA twin pregnancies complicated by TTTS and undergoing FLS in women with a short CL, while the level of evidence for intervention with vaginal progesterone is insufficient for evaluation. However, the small sample sizes of the included studies, lack of comparison in the original publications and lack of stratification of the observed outcomes according to Quintero stage, gestational age at FLS and CL cut‐off highlight the need for appropriately powered studies. © 2025 The Author(s). *Ultrasound in Obstetrics & Gynecology* published by John Wiley & Sons Ltd on behalf of International Society of Ultrasound in Obstetrics and Gynecology.

## INTRODUCTION

Preterm birth (PTB) is the leading cause of perinatal mortality and morbidity worldwide, with an estimated 15 million PTBs occurring every year. Despite all efforts, PTB remains the leading cause of mortality in children under the age of 5 years and the main cause of over half of perinatal‐related long‐term morbidity^1^. PTB is more common in twin pregnancy, with studies reporting an incidence of up to 50% in twin pregnancies delivered < 37 weeks, 20% < 34 weeks and 10% < 32 weeks^2^, ^3^, ^4^. The risk of PTB varies according to chorionicity and amnionicity, which could be explained by the higher rate of complications among monochorionic twin pregnancies^5^, ^6^, ^7^, ^8^. One of the risks is the development of twin‐to‐twin transfusion syndrome (TTTS), which complicates more than 10% of all monochorionic twin pregnancies^9^, ^10^. TTTS occurs due to unbalanced transfusion across placental vascular connections^11^. It is associated with a significant increase in perinatal mortality and morbidity, with a mortality rate of up to 90% in untreated advanced cases^12^, ^13^.

In cases of TTTS, many factors contribute to pregnancy outcome, especially the occurrence of PTB^10^, ^14^, ^15^. Prematurity was reported to be an independent predictor of neurodevelopmental impairment in cases of TTTS^16^, ^17^. One of the primary diagnostic criteria of TTTS is polyhydramnios around the recipient twin, which can increase the risk of PTB due to uterine overdistension, which causes cervical shortening^18^. The early stages of TTTS are usually managed conservatively, whereas advanced stages are commonly managed with fetoscopic laser photocoagulation of the placental anastomoses. However, surgical intervention in combination with underlying polyhydramnios increases the risk of preterm labor^19^.

Evidence on the benefits of cervical cerclage for prevention of PTB in twin pregnancy is accumulating^19^, ^20^. Other interventional measures, such as vaginal progesterone and cervical pessary, have also been evaluated^19^. However, studies have reported conflicting results. We conducted a systematic review to assess the available evidence on interventional measures to prevent PTB following fetoscopic laser surgery (FLS) in monochorionic diamniotic (MCDA) twin pregnancies complicated by TTTS.

## METHODS

### Protocol, information sources and literature search

This systematic review was performed according to a protocol designed *a priori* that is recommended for systematic reviews and meta‐analyses^20^, ^21^, ^22^. The MEDLINE, Embase and Cochrane databases were searched electronically from inception to November 2023, followed by an update in February 2024, using a combination of the relevant medical subject heading (MeSH) terms, keywords and word variants for ‘twin‐to‐twin transfusion syndrome’, ‘preterm birth’ and ‘outcome’ (Table S1). The search and selection criteria were restricted to the English language. Reference lists of relevant articles and reviews were searched manually for additional reports. The Preferred Reporting Items for Systematic Reviews and Meta‐Analyses (PRISMA) guidelines were followed^23^. The study was registered with the Prospero database (registration number: CRD42023444949).

### Outcomes measures, study selection and data collection

The inclusion criteria were studies on MCDA twin pregnancies undergoing FLS for TTTS, comparing those receiving with those not receiving a preventive strategy for reducing the risk of PTB. The primary outcome was gestational age at birth. The secondary outcomes were the interval between FLS and birth, PTB < 34 weeks, PTB < 32 weeks, PTB < 28 weeks, PTB < 24 weeks, delivery within 2 weeks after FLS, delivery within 4 weeks after FLS, preterm prelabor rupture of membranes (PPROM), chorioamnionitis, double survival (defined as survival of both twins), no survival (defined as death of both twins before birth), survival of at least one twin, overall fetal or perinatal loss (defined as the sum of miscarriage, intrauterine death and neonatal death), and overall fetal or perinatal survival. Furthermore, we aimed to compare the observed outcomes according to the Quintero stage of TTTS and gestational age at FLS.

Studies including twin pregnancies with structural or chromosomal anomalies and studies from which data on the observed outcomes could not be extrapolated were excluded. Studies published before 2000 were also excluded because we considered that advances in prenatal imaging techniques and improvements in the diagnosis and treatment of TTTS made them less relevant. Conference abstracts and case series with fewer than five cases were excluded to avoid publication bias.

Two authors (F.D.A. and N.E.) reviewed all abstracts independently. Agreement regarding potential relevance was reached by consensus. Full‐text copies of articles deemed relevant were obtained, and the same two authors independently extracted relevant data regarding study characteristics and pregnancy outcomes. Inconsistencies were resolved through discussion among the reviewers until consensus was reached or through discussion with a third author (A.K.). If more than one study had been published for the same cohort with identical endpoints, the report containing the most comprehensive information on the population was included in the pooled data analysis to avoid overlapping populations.

### Quality assessment and risk of bias

Risk of bias for randomized controlled trials (RCTs) was assessed using the Revised Cochrane risk‐of‐bias tool for randomized trials (RoB 2)^24^. According to this tool, the risk of bias of each included study is judged in five domains: bias arising from the randomization process, bias due to deviations from intended interventions, bias due to missing outcome data, bias in the measurement of the outcome and bias in the selection of the reported result. Although the RoB 2 tool does not provide an overall risk of bias assessment, the risk of bias was considered low if four or more domains were rated as low risk (not including ‘other biases’), with at least one being bias arising from the randomization process, according to that reported in previous systematic reviews of intervention^24^.

Risk of bias for observational studies was evaluated using the Risk Of Bias In Non‐randomized Studies of Interventions (ROBINS‐I) tool^25^. ROBINS‐I provides a detailed framework for the assessment and judgement of risk of bias that may arise due to confounding, selection of participants, measurement of interventions, missing data, measurement of outcomes and selection of reported results^25^. The ROBINS‐I tool is equally appropriate for use in cross‐sectional and longitudinal non‐randomized studies because quality assessment is independent of study design. Each domain is determined to exhibit low, moderate, serious or critical risk of bias. Low risk indicates that the study is ‘comparable to a well‐performed randomized trial’ in the domain being evaluated. Moderate risk of bias indicates that the study is ‘sound for a non‐randomized study’, but not comparable to a rigorous randomized trial. Serious risk of bias indicates the presence of ‘important problems’, while critical risk of bias indicates the study is ‘too problematic’ to provide any useful evidence on the effect of intervention. If insufficient information is provided to determine the risk of bias of a certain domain, the domain is marked as having no information.

The Grading of Recommendations, Assessment, Development and Evaluation (GRADE) methodology was used to assess the quality of the retrieved evidence (GRADEpro, Version 20, 2014, McMaster University, Hamilton, ON, Canada)^26^.

### Statistical analysis

For categorical outcomes, random‐effects meta‐analysis of proportions was used to combine data and results are reported as odds ratio (OR) (95% CI). For continuous variables, random‐effects pooled mean difference was used to combine the data and results are reported as mean difference (95% CI). Funnel plots displaying the outcome rate from individual studies *vs* their precision (1/standard error) were carried out with an exploratory aim. Tests for funnel‐plot asymmetry were not used because the total number of publications included for each outcome was less than 10. In this case, the power of the test is too low to distinguish chance from real asymmetry^27^, ^28^.

Between‐study heterogeneity was explored using the *I* ^2^ statistic, which represents the percentage of between‐study variation that is due to heterogeneity rather than chance^27^. A value of 0% indicates no observed heterogeneity, whereas *I* ^2^ values of ≥ 50% indicate a substantial level of heterogeneity. All analyses were performed using StatsDirect Statistical software (StatsDirect Ltd, Cambridge, UK) and Comprehensive Meta‐analysis software version 4 (Biostat, Englewood, NJ, USA).

## RESULTS

### Study selection and characteristics

**Figure 1:**
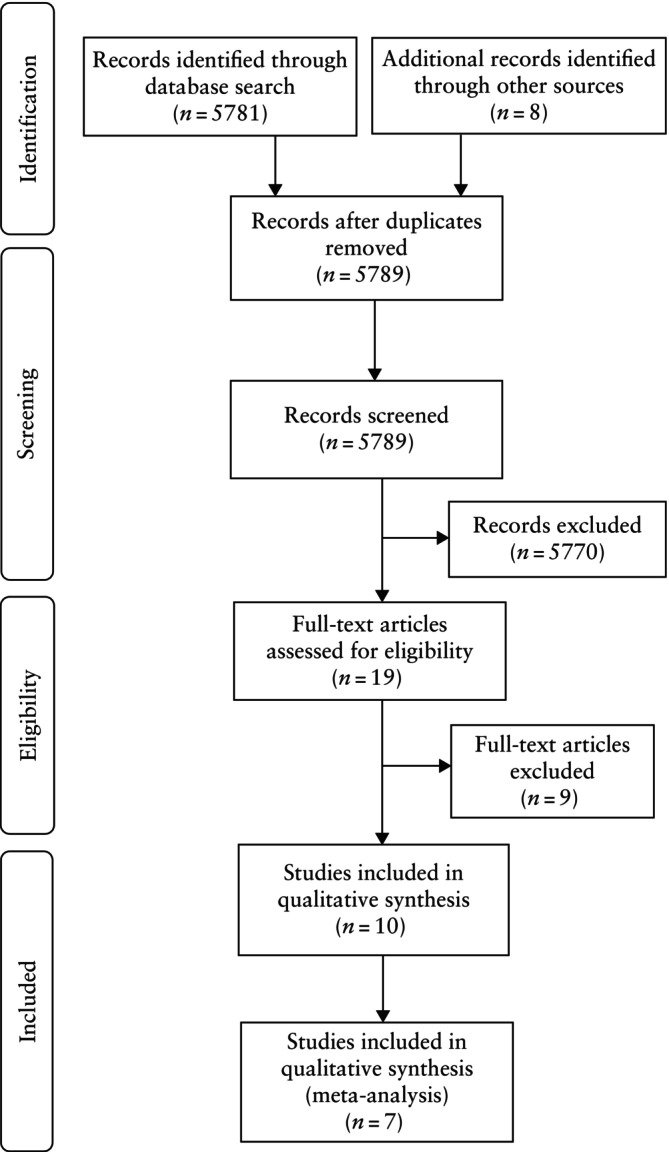
Flowchart summarizing inclusion of studies in systematic review and meta‐analysis.

A total of 5789 articles were identified, of which 19 were assessed with respect to their eligibility for inclusion and 10 of these studies were included in the systematic review (Tables 1 and S2, Figure 1)^29^, ^30^, ^31^, ^32^, ^33^, ^34^, ^35^, ^36^, ^37^, ^38^. These 10 studies included 1159 MCDA twin pregnancies complicated by TTTS that underwent FLS.

**Table 1:** General characteristics of studies included in systematic review reporting on monochorionic diamniotic twin pregnancies complicated by twin‐to‐twin transfusion syndrome and undergoing fetoscopic laser surgery (FLS), comparing those receiving *vs* those not receiving intervention to reduce risk of preterm birth (PTB)

Study	Country	Study design	Study period	Intervention	Criteria for intervention	Primary outcome	Pregnancies (*n*)
Rodo (2023)^29^	Spain, Belgium, Germany	Multicenter open‐label RCT	2012–2019	Pessary	All pregnancies undergoing FLS	PTB < 32 weeks	137
Bartin (2024)^30^	France, Brazil	Retrospective	2013–2022	Cerclage, pessary	CL < 25 mm	Delivery within 2 weeks or 1 month after FLS	134
Buskmiller (2022)^31^	USA	Prospective multicenter	2013–2020	Pessary, cerclage, progesterone, combined treatment	CL < 30 mm	Interval between FLS and delivery	255
Mustafa (2022)^32^	USA	Retrospective multicenter	2012–2018	Indomethacin	All pregnancies undergoing FLS	GA at delivery	411
Ruegg (2020)^33^	Switzerland	Retrospective single center	2008–2014	Cerclage	CL < 25 mm	Survival rate of at least one infant to 6 months of age	10
Carreras (2012)^34^	Spain	Retrospective single center	2007–2010	Pessary	CL ≤ 25 mm	GA at delivery	16
Papanna (2012)^35^	USA	Retrospective multicenter	2004–2010	Cerclage	CL ≤ 25 mm	GA at delivery and perinatal mortality	163
Cobo (2011)^36^	Spain	Prospective single center	2006–2008	Cerclage	CL ≤ 15 mm	PTB within 7 days after FLS	10
Salomon (2008)^37^	France	Retrospective single center	1999–2007	Cerclage	CL < 15 mm	GA at delivery and perinatal outcome	14
Robyr (2005)^38^	France, Belgium, Germany, Switzerland	Retrospective multicenter	2002–2003	Cerclage	CL < 20 mm	GA at delivery	9

Only first author is given for each study. CL, cervical length; GA, gestational age; RCT, randomized controlled trial.

Only one RCT^29^ was included in this systematic review, which was scored as high risk of bias according to the RoB 2 tool (Table S3). The study was carried out in four European centers and included 137 MCDA twin pregnancies complicated by TTTS, randomized to either cervical pessary or no intervention, but was stopped because the futility analysis after the first interim analysis resulted in a conditional probability of <1% of achieving the primary outcome^29^. Of the nine observational studies included in the present systematic review, five reported cerclage only, one reported pessary only, one reported cerclage and pessary, one reported pessary, cerclage, progesterone and combined treatment, and one reported indomethacin (Table 1). Three studies were not included in the pooled data analysis^32^, ^36^, ^38^. The study of Mustafa *et al*.^32^ was the only study that reported the use of only indomethacin to reduce the risk of PTB after FLS, thus no pooled data synthesis could be performed. In the study of Cobo *et al*.^36^, the authors included 12 women who underwent cervical cerclage after FLS, 10 of whom had preoperative cervical length (CL) ≤ 15 mm. They reported that, in all of these cases, the CL increased postoperatively, but they did not state the gestational age at birth or the risk of PTB in this group and the no intervention group. Finally, in the study of Robyr *et al*.^38^, the authors reported that nine patients had a CL ≤ 20 mm and all patients received cerclage, thus we could not compute the risk of PTB in women who did not receive cerclage^38^.

Assessment of risk of bias of observational studies according to the ROBINS‐I tool is presented in Table 2. All studies but one were at moderate or serious risk of bias. Four studies^33^, ^36^, ^37^, ^38^ were downgraded from moderate to serious or critical risk of bias due to the heterogeneous inclusion criteria, outcome assessment and threshold for intervention (Table 2).

**Table 2:** Quality assessment of observational studies according to Risk Of Bias In Non‐randomized Studies of Interventions (ROBINS‐I) tool

Study	Confounding	Selection	Measurement of intervention	Missing data	Measurements of the outcome	Reported results	Overall
Bartin (2024)^30^	Critical	Moderate	Moderate	Low	Moderate	Low	Moderate
Buskmiller (2022)^31^	Low	Moderate	Moderate	Low	Low	Low	Moderate
Mustafa (2022)^32^	Moderate	Moderate	Moderate	Moderate	Moderate	Moderate	Moderate
Ruegg (2020)^33^	Serious	Moderate	Low	Moderate	Serious	Serious	Serious
Carreras (2012)^34^	Moderate	Moderate	Low	Moderate	Moderate	Low	Moderate
Papanna (2012)^35^	Low	Moderate	Moderate	Low	Low	Low	Moderate
Cobo (2011)^36^	Critical	Serious	Moderate	Serious	Serious	Moderate	Serious
Salomon (2008)^37^	Serious	Moderate	Serious	Moderate	Moderate	Moderate	Serious
Robyr (2005)^38^	Critical	Critical	Critical	Moderate	Moderate	Serious	Critical

Only first author is given for each study. Risk of bias: low, study is comparable to well‐performed randomized trial regarding this domain; moderate, study is sound for non‐randomized study with regard to this domain, but cannot be considered comparable to well‐performed randomized trial; serious, study has some important problems in this domain; critical, study is too problematic in this domain to provide any useful evidence on effects of intervention.

### Synthesis of results

**Figure 2:**
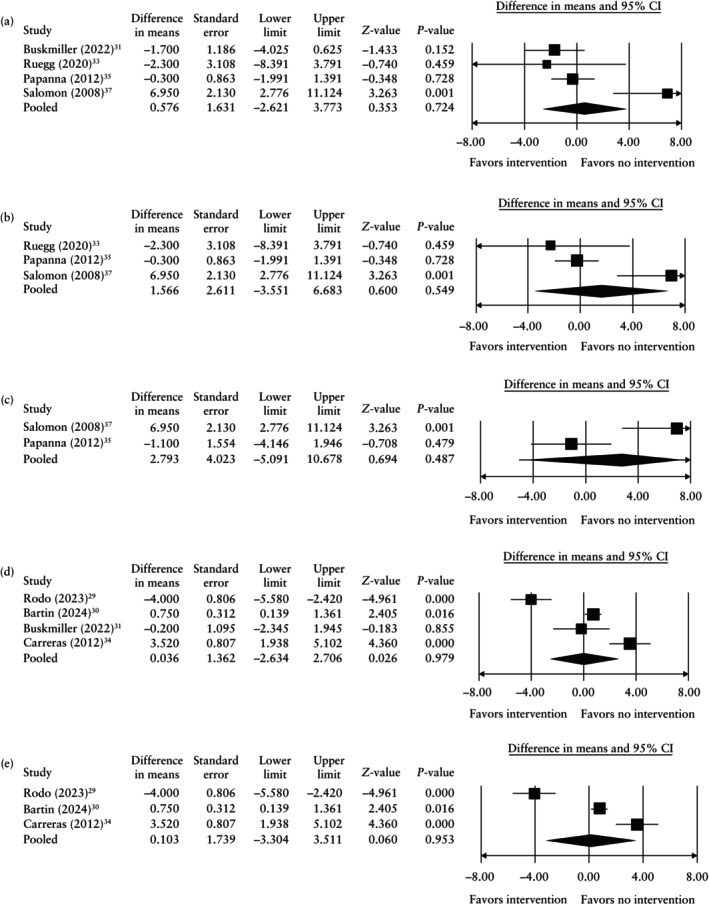
Forest plots of pooled mean differences (95% CI) for gestational age at birth in monochorionic diamniotic twin pregnancies complicated by twin‐to‐twin transfusion syndrome receiving cervical cerclage with cervical length (CL) < 30 mm (a), CL < 25 mm (b) or CL < 15 mm (c), or cervical pessary with CL < 30 mm (d) or CL < 25 mm (e), compared with no intervention. Only first author is given for each study.

There was no significant difference in mean gestational age at birth in MCDA twin pregnancies undergoing FLS for TTTS in women receiving *vs* those not receiving cervical cerclage with CL < 30 mm (*P* = 0.724), < 25 mm (*P* = 0.549), < 20 mm (*P* = 0.387) or < 15 mm (*P* = 0.487) (Figure 2a–c and Table 3). Similarly, intervention with cervical pessary was not associated with a higher gestational age at birth compared with no intervention in MCDA twin pregnancies in women with CL < 30 mm (*P* = 0.979) or < 25 mm (*P* = 0.953) (Figure 2d,e). There was also no significant difference in the mean interval between FLS and delivery in women receiving *vs* those not receiving cervical cerclage with CL < 30 mm (*P* = 0.285), < 25 mm (*P* = 0.282), < 20 mm (*P* = 0.756) and < 15 mm (*P* = 0.238), and in those receiving *vs* those not receiving cervical pessary with CL < 30 mm (*P* = 0.547) and < 25 mm (*P* = 0.093) (Table 3). It was not possible to perform pooled data synthesis comparing gestational age at birth or interval between FLS and delivery in women receiving progesterone or other pharmacological therapy with those who received no intervention.

**Table 3:** Pooled mean differences for different outcomes in monochorionic diamniotic twin pregnancies complicated by twin‐to‐twin transfusion syndrome and undergoing fetoscopic laser surgery (FLS), according to preterm birth prevention strategy

Outcome	Studies (*n* ^ref^)	Intervention (*n*)	No intervention (*n*)	Mean difference* (95% CI)	*I* ^2^ (%)	*P*
*Cervical cerclage vs no intervention*						
CL < 30 mm						
GA at birth (weeks)	4^31,33,35,37^	115	244	0.58 (−2.62 to 3.73)	34.2	0.724
Interval from FLS to delivery (weeks)	3^31,35,37^	109	240	3.95 (−3.30 to 11.20)	72.4	0.285
CL < 25 mm						
GA at birth (weeks)	3^33,35,37^	94	93	1.57 (−3.55 to 6.68)	8.7	0.549
Interval from FLS to delivery (weeks)	2^35,37^	88	89	3.98 (−3.27 to 11.23)	78.1	0.282
CL < 20 mm						
GA at birth (weeks)	3^33,35,37^	94	93	2.08 (−2.63 to 6.79)	14.1	0.387
Interval from FLS to delivery (weeks)	2^35,37^	88	89	1.84 (−9.73 to 13.41)	90.0	0.756
CL < 15 mm						
GA at birth (weeks)	2^35,37^	58	27	2.79 (−5.09 to 10.68)	0	0.487
Interval from FLS to delivery (weeks)	2^35,37^	58	27	4.21 (−2.78 to 11.19)	89.1	0.238
*Cervical pessary vs no intervention*						
CL < 30 mm						
GA at birth (weeks)	4^29,30,31,34^	89	228	0.04 (−2.63 to 2.71)	23.1	0.979
Interval from FLS to delivery (weeks)	4^29,30,31,34^	89	228	0.36 (−0.33 to 1.57)	72.8	0.547
CL < 25 mm						
GA at birth (weeks)	3^29,30,34^	68	77	0.10 (−3.30 to 3.51)	36.0	0.953
Interval from FLS to delivery (weeks)	3^29,30,34^	68	77	15.34 (−2.56 to 20.24)	65.9	0.093

* Direction of difference is intervention – no intervention. CL, cervical length; GA, gestational age.

Computation of the comparison of categorical outcomes between MCDA twin pregnancies receiving *vs* those not receiving intervention was affected by the fact that most studies did not report a control population, thus the results were first reported as pooled proportions in the population of MCDA twin pregnancies receiving the intervention.

In women with CL < 30 mm, cervical cerclage was associated with an increased risk of PTB < 32 weeks (OR, 5.20 (95% CI, 2.17–12.46)) and < 28 weeks (OR, 3.48 (95% CI, 1.74–6.99)) compared to no intervention, although the analysis was based on only 42 pregnancies receiving cervical cerclage and included two studies reporting different cut‐offs for intervention (30 mm and 25 mm, respectively) (Table 4). Cervical cerclage was not associated with a significant reduction in the risk of PTB < 24 weeks (*P* = 0.331), PPROM (*P* = 0.572), delivery within 2 weeks (*P* = 0.469) or 4 weeks (*P* = 0.212) after FLS, or perinatal loss (*P* = 0.089) compared with no intervention (Table 4).

**Table 4:** Pooled odds ratio (OR) for different outcomes in monochorionic diamniotic twin pregnancies complicated by twin‐to‐twin transfusion syndrome and undergoing fetoscopic laser surgery (FLS), according to preterm birth (PTB) prevention strategy

Outcome	Studies (*n* ^ref^)	Cases (*n/N vs n/N*)	Pooled OR (95% CI)	*I* ^2^ (%)	*P*
*Cervical cerclage* vs *no intervention*					
CL < 30 mm					
PTB < 32 weeks	2^30,31^	35/42 *vs* 116/212	5.20 (2.17–12.46)	0	0.002
PTB < 28 weeks	2^30,31^	24/42 *vs* 62/212	3.48 (1.74–6.99)	0	0.004
PTB < 24 weeks	2^30,31^	11/42 *vs* 26/212	2.91 (0.34–25.07)	0	0.331
Delivery within 4 weeks after FLS	2^30,31^	17/42 *vs* 54/212	1.96 (0.68–5.63)	59.2	0.212
Delivery within 2 weeks after FLS	2^30,31^	2/42 *vs* 36/212	1.88 (0.34–10.34)	75.2	0.469
Overall fetal or perinatal loss	2^30,31^	34/84 *vs* 124/396	1.65 (0.93–2.95)	26.2	0.089
Overall fetal or perinatal survival	2^30,31^	50/84 *vs* 272/396	0.60 (0.39–1.08)	26.2	0.089
PPROM	3^31,35,37^	41/105 *vs* 68/226	1.40 (0.44–4.46)	65.3	0.572
CL < 25 mm					
Survival of both fetuses	2^30,37^	66/100 *vs* 90/145	0.99 (0.29–3.31)	75.5	0.982
Survival of ≥ 1 fetus	2^30,37^	84/100 *vs* 15/145	0.95 (0.14–7.09)	86.9	0.988
No fetal survival	2^30,37^	16/100 *vs* 30/145	1.02 (0.14–7.31)	86.9	0.988
Overall fetal or perinatal loss	3^30,37^	71/218 *vs* 91/300	0.82 (0.26–2.60)	81.6	0.736
Overall fetal or perinatal survival	3^30,35,37^	147/218 *vs* 209/300	1.22 (0.38–3.87)	81.6	0.736
PPROM	2^30,37^	31/88 *vs* 27/89	0.59 (0.06–6.06)	69.2	0.660
Chorioamnionitis	2^35,37^	2/88 *vs* 4/89	0.47 (0.07–3.00)	0	0.426
*Cervical pessary* vs *no intervention*					
CL < 30 mm					
PTB < 32 weeks	3^29,30,31^	46/81 *vs*119/220	1.56 (0.87–2.79)	63.0	0.132
PTB < 28 weeks	2^30,31^	20/73 *vs* 62/212	1.14 (0.60–2.17)	0	0.686
PTB < 24 weeks	2^30,31^	12/73 *vs* 26/212	1.85 (0.78–4.38)	0	0.162
Delivery within 4 weeks after FLS	2^30,31^	17/73 *vs* 54/212	1.10 (0.29–4.14)	74.6	0.889
Delivery within 2 weeks after FLS	2^30,31^	11/73 *vs* 36/212	1.05 (0.48–2.27)	0	0.910
Overall fetal or perinatal loss	3^29,30,31^	46/160 *vs* 33/412	0.96 (0.36–2.57)	75.4	0.233
Overall fetal or perinatal survival	3^29,30,31^	114/160 *vs* 279/412	1.04 (0.39–2.79)	75.4	0.233
Survival of both fetuses	3^29,30,31^	58/80 *vs* 158/206	1.25 (0.66–2.40)	0	0.492
Survival of ≥ 1 fetus	3^29,30,31^	75/80 *vs* 180/206	2.46 (0.12–50.65)	90.4	0.556
No fetal survival	3^29,30,31^	7/80 *vs* 19/206	1.16 (0.41–3.29)	28.9	0.774
PPROM	4^29,30,31,34^	13/89 *vs* 66/214	0.52 (0.17–1.56)	46.5	0.241
CL < 25 mm					
PTB < 32 weeks	2^29,30^	33/60 *vs* 26/69	2.00 (0.98–4.06)	0	0.056
PPROM	3^29,30,31^	8/68 *vs* 25/77	0.50 (0.09–2.79)	49.2	0.224
Overall fetal or perinatal loss	3^29,30,31^	24/136 *vs* 39/154	0.63 (0.35–1.13)	0	0.122
Overall fetal or perinatal survival	3^29,30,31^	112/136 *vs* 115/154	1.59 (0.88–2.84)	0	0.122
Survival of both fetuses	2^29,30^	45/60 *vs* 49/69	1.19 (0.54–2.60)	0	0.670
Survival of ≥ 1 fetus	2^29,30^	56/60 *vs* 52/69	16.87 (0.13–20.68)	81.7	0.256
No fetal survival	2^29,30^	6/60 *vs* 10/69	0.69 (0.24–1.96)	0	0.484

CL, cervical length; PPROM, preterm prelabor rupture of membranes.

In women with CL < 25 mm, cervical cerclage after FLS was not associated with a reduced risk of PPROM (*P* = 0.660), survival of at least one fetus (*P* = 0.988) or perinatal loss (*P* = 0.736) compared with no intervention (Table 4).

In women with CL < 30 mm, cervical pessary was not associated with a reduced risk of PTB < 32 weeks (*P* = 0.132), < 28 weeks (*P* = 0.686) or < 24 weeks (*P* = 0.162), delivery within 2 weeks (*P* = 0.910) or 4 weeks (*P* = 0.889) after FLS, or perinatal loss (*P* = 0.233) compared with no intervention. Likewise, in women with CL < 25 mm, cervical pessary did not reduce the risk of PTB < 32 weeks (*P* = 0.056), PPROM (*P* = 0.224) or perinatal loss (*P* = 0.122).

Assessment of the quality of retrieved evidence according to GRADE is presented in Figure S1. Overall, the certainty of evidence was very low in showing that cervical cerclage or pessary could affect gestational age at birth in MCDA twin pregnancies that underwent FLS for TTTS, irrespective of the degree of cervical shortening. The level of evidence was downgraded to very low due to the high risk of bias of most of the included studies and because of the serious or very serious inconsistency, indirectness and imprecision of the studies.

## DISCUSSION

### Summary of main findings

The findings of this study demonstrate that there is no evidence supporting the use of cervical cerclage or cervical pessary to reduce the risk of PTB in women undergoing FLS for TTTS. It was not possible to draw any conclusion on intervention using vaginal progesterone, although the only study exploring its role after FLS for TTTS did not report any benefit in preventing PTB or improving perinatal outcome. The small sample sizes of the included studies, lack of comparison in the original publications and lack of stratification of the observed outcomes according to Quintero stage of TTTS, gestational age at FLS and CL cut‐off do not allow for extrapolation of objective evidence on the role of preventive strategies for PTB after FLS, especially in women presenting with short CL.

### Strengths and limitations

This systematic review and meta‐analysis is the first to attempt to quantify the evidence on the prevention of PTB in MCDA twin pregnancies complicated by TTTS and undergoing FLS. The small number of cases in some of the included studies, their retrospective non‐randomized design and lack of standardized criteria for prenatal surveillance and timing of delivery represent the major limitations of this systematic review. Most of the included studies were retrospective observational studies reporting the outcome of pregnancies that received a given intervention for PTB after undergoing FLS, without reporting the corresponding values in a control population. In this scenario, computation of the risk is not feasible. We also could not report the observed outcomes according to the TTTS stage, gestational age at FLS, maternal symptoms and CL prior to FLS. Another major limitation of the present review is the paucity of data and lack of stratification according to different CL cut‐offs in women receiving cervical pessary, and it was not possible to perform any pooled data analysis on the use of progesterone, thus making meta‐analysis for this intervention unattainable.

### Clinical and research implications

PTB is the leading cause of perinatal mortality and morbidity in multiple pregnancy and this risk further increases when pregnancy is complicated by TTTS. However, the role of preventive strategies for PTB in multiple pregnancy is more controversial^39^, ^40^, ^41^. The role of cervical cerclage in preventing PTB in multiple pregnancy has also been revised recently. Previous studies published more than two decades ago did not report any beneficial effect of cervical cerclage in reducing the risk of PTB or improving perinatal outcomes^42^, ^43^. More recent studies have highlighted the potential beneficial role of cervical cerclage in prolonging twin pregnancy in women presenting with cervical dilatation or short CL. A RCT by Roman *et al*.^19^ including twin pregnancies presenting with asymptomatic cervical dilatation before 24 weeks' gestation reported that a combination of physical examination‐indicated cerclage, indomethacin and antibiotics decreased PTB < 28 weeks' gestation by 50%, with a 78% decrease in perinatal mortality. A more recent meta‐analysis^44^ confirmed these findings, reporting a significant reduction in PTB and adverse perinatal outcomes in women with a twin pregnancy presenting with asymptomatic cervical dilatation or CL < 15 mm on ultrasound. In the present study, cervical cerclage did not affect gestational age at birth or reduce the risk of PTB or perinatal loss in women with a MCDA twin pregnancy undergoing FLS, but CL < 30 mm was associated with an increased risk of PTB < 32 and < 28 weeks. However, the analysis was based on a limited number of pregnancies receiving cervical cerclage and included two studies reporting different CL cut‐offs for intervention (30 mm and 25 mm, respectively), thus suggesting a spurious association.

In the present study, cervical pessary was not associated with a higher gestational age at birth or a reduced risk of PTB or perinatal survival. There are no robust data on the use of cervical pessary to prevent PTB in twin pregnancy and the evidence in singleton pregnancy is controversial^45^, ^46^. Some investigators have suggested that pessaries may predispose the patient to PPROM by creating a dysbiotic vaginal microbiome. The PECEP LASER RCT^29^, which aimed to assess whether cervical pessary prolongs pregnancy in cases of TTTS regardless of CL at the time of FLS, was terminated prematurely as the results of the interim analysis did not favor study continuation^29^. A recent RCT on the use of cervical pessary in twin pregnancies with a short CL, the STOPPIT 2 trial^47^, did not show a beneficial effect of pessary in reducing the risk of PTB or adverse perinatal outcome, and the use of pessary in twin pregnancy is generally not recommended by professional societies.

Despite the high prevalence of PTB in pregnancies complicated by TTTS and the potential beneficial effect of either vaginal progesterone or cervical cerclage, the findings from this systematic review did not show any role of such strategies in reducing the risk of PTB in these women, other than a spurious finding for cervical cerclage. The lack of effect may be explained on several bases. First, the included studies were retrospective observational studies with small sample sizes, thus making most of these studies underpowered. Second, there were significant differences in potential confounders among the included studies. Third, none of the included studies reported subgroup analyses according to gestational age at FLS or TTTS stage. This is crucial because the increasing severity of polyhydramnios associated with the most advanced stages of TTTS may account for a higher risk of PTB, irrespective of the choice of therapeutic strategy.

Most studies on preventive strategies for PTB collect limited observational data and it has been suggested that progesterone, cerclage and pessary may have different effects depending on the phenotype of PTB and patient characteristics. The findings from this systematic review can serve as a basis to design appropriately powered studies comparing the different preventive strategies for PTB in women undergoing FLS for TTTS. Ideally, future studies should include asymptomatic twin pregnancies with no other risk factors for PTB, stratified according to TTTS stage and gestational age at FLS, and evaluate relevant obstetrical outcomes such as PPROM and gestational age at birth.

### Conclusions

Current evidence does not support that cervical cerclage or pessary may prolong gestation or reduce the risk of PTB in pregnancies undergoing FLS for TTTS and presenting with short CL. Furthermore, there is insufficient evidence regarding the role of progesterone in affecting these outcomes, although the only study exploring its use in pregnancies complicated by TTTS did not report any beneficial effect. The findings from this systematic review highlight the need for appropriately powered studies aimed at exploring the role of the most common preventive strategies for PTB in women undergoing FLS for TTTS.

## Supporting information


**Table S1** Search strategy

**Table S2** Excluded studies and reason for exclusion

**Table S3** Risk of bias of randomized controlled trial, assessed using Revised Cochrane risk‐of‐bias tool for randomized trials (RoB 2)

**Table S4** Pooled proportions (95% CI) for different outcomes explored in systematic review in monochorionic diamniotic twin pregnancies complicated by twin‐to‐twin transfusion syndrome undergoing fetoscopic laser surgery, according to type of preterm birth prevention strategy

**Figure S1** Grading of Recommendations, Assessment, Development and Evaluation (GRADE) methodology for primary outcome.

## Data Availability

N/A.
