# Supplementary material for: Interventions to prevent preterm birth following fetoscopic laser surgery for twin‐to‐twin transfusion syndrome: systematic review and meta‐analysis
Source: Ultrasound Obstet Gynecol. 2025 Jun 5;66(1):14–23. doi: 10.1002/uog.29230 (PMC12209700; doi:10.1002/uog.29230)
Supplement: Supplementary file 1 — Table S1 Search strategy [file UOG-66-14-s005.docx]

**Search Strategy**

**Databases:** Embase®, MEDLINE® (date run: 24 November 2022)

| **Set#** | **Searched for** | **Results** |
| --- | --- | --- |
| S1 | MESH.EXACT("Fetofetal Transfusion") | 2308° |
| S2 | EMB.EXACT.EXPLODE("twin twin transfusion syndrome") | 3185° |
| S3 | ti,ab,if("twin twin" or "twin to twin" or ttts or ((twin or intertwin or fetofetal or "feto fetal" or foetofoetal or "foeto foetal" or "intrauterine cross" or "intra uterine cross") near/5 transfusion)) | 7514* |
| S4 | ti,ab,if("twin anaemia polycythaemia" or "twin anemia polycythemia" or "twin anaemia polythaemia" or "twin anemia polythemia" "twin oligohydramnios polyhydramnios" or "twin oligopolyhydramnios" or "twin oligo polyhydramnios") | 345° |
| S5 | ti,ab,if((taps or tops) and (twin or twins)) | 290° |
| S6 | s1 or s2 or s3 or s4 or s5 | 7774* |
| S7 | (s1 or s2 or s3 or s4 or s5) and pd(2016-2023) | 1668° |
| **S8** | **(s1 or s2 or s3 or s4 or s5) and dcre(20221012-20221124)** | **39°** |
| S9 | s6 not (s7 or s8) | 5334* |
| **S10** | **s9 and pd(2000-2015)** | **2682°** |
| **S11** | **s9 not s10** | **812°** |

* Duplicates are removed from the search, but included in the result count.

° Duplicates are removed from the search and from the result count.

**Database:** The Cochrane Library (date run: 24 November 2022)

| **ID** | **Search** | **Hits** |
| --- | --- | --- |
| #1 | MeSH descriptor: [Fetofetal Transfusion] this term only | 25 |
| #2 | ("twin twin" or "twin to twin" or ttts):ti,ab,kw (Word variations have been searched) | 101 |
| #3 | ((twin or intertwin or fetofetal or "feto fetal" or foetofoetal or "foeto foetal" or "intrauterine cross" or "intra uterine cross") near/5 transfusion):ti,ab,kw (Word variations have been searched) | 94 |
| #4 | ("twin anaemia polycythaemia" or "twin anemia polycythemia" or "twin anaemia polythaemia" or "twin anemia polythemia" "twin oligohydramnios polyhydramnios" or "twin oligopolyhydramnios" or "twin oligo polyhydramnios"):ti,ab,kw (Word variations have been searched) | 14 |
| #5 | ((taps or tops) and (twin or twins)):ti,ab,kw (Word variations have been searched) | 34 |
| **#6** | **#1 or #2 or #3 or #4 or #5** | **128** |

- The Cochrane Database of Systematic Reviews (CDSR, Cochrane Reviews: Issue 11 of 12, November 2022) (6 references)
- The Cochrane Central Register of Controlled Trials (CENTRAL, Trials: Issue 10 of 12, October 2022) (122 references)

The references in set #6 were supplied in the search results emailed to you on 13 October 2022 and have not been downloaded again for this document because the final results hit in set #6 remains unchanged.
