# Supplementary material for: Interventions to prevent preterm birth following fetoscopic laser surgery for twin‐to‐twin transfusion syndrome: systematic review and meta‐analysis
Source: Ultrasound Obstet Gynecol. 2025 Jun 5;66(1):14–23. doi: 10.1002/uog.29230 (PMC12209700; doi:10.1002/uog.29230)
Supplement: Supplementary file 2 — Table S2 Excluded studies and reason for exclusion [file UOG-66-14-s001.docx]

**Table S2** Excluded studies and reason for exclusion

| **Author** | **Year** | **Title** | **Reason for the exclusion** |
| --- | --- | --- | --- |
| MALSHE | 2017 | Preterm delivery after fetoscopic laser surgery for twin–twin  transfusion syndrome: etiology and risk factors | Did not include details about PTB measures. |
| Rodó | 2017 | Arabin cervical pessary for prevention of preterm birth in cases of twin-to-twin transfusion syndrome treated by fetoscopic  LASER coagulation: the PECEP LASER randomised controlled trial | Study protocol |
| Groussolles | 2019 | Neonatal Outcomes and Maternal Characteristics in Monochorionic Diamniotic Twin Pregnancies:  Uncomplicated versus Twin-to-Twin Transfusion  Syndrome Survivors after Fetoscopic Laser Surgery | Did not include details about PTB measures. |
| Valenzuela | 2020 | Foetal therapies and their influence on preterm birth | Review, No original data included |
| Zijl | 2017 | Pessary or Progesterone to Prevent Preterm delivery in women with short cervical length: the Quadruple P randomised controlled trial | Study protocol |
| Stirnemann | 2019 | Preterm premature rupture of membranes is a collateral effect of improvement in perinatal outcomes following fetoscopic coagulation of chorionic vessels for twin–twin transfusion  syndrome: a retrospective observational study of 1092 cases | Did not include details about PTB measures. |
| Chmait | 2013 | Perioperative characteristics associated with preterm birth in  twin-twin transfusion syndrome treated by laser surgery | No available data for the cerclage group |
