# Supplementary material for: Interventions to prevent preterm birth following fetoscopic laser surgery for twin‐to‐twin transfusion syndrome: systematic review and meta‐analysis
Source: Ultrasound Obstet Gynecol. 2025 Jun 5;66(1):14–23. doi: 10.1002/uog.29230 (PMC12209700; doi:10.1002/uog.29230)
Supplement: Supplementary file 3 — Table S3 Risk of bias of randomized controlled trial, assessed using Revised Cochrane risk‐of‐bias tool for randomized trials (RoB 2) [file UOG-66-14-s004.docx]

**Table S3** Risk of bias of randomized controlled trial, assessed using Revised Cochrane risk-of-bias tool for randomized trials (RoB 2)

| Study | Randomization process | Deviations from intended interventions | Mising outcome data | Measurement of the outcome | Selection of the reported result | Overall Bias |
| --- | --- | --- | --- | --- | --- | --- |
| Rodo^29^ | Low risk | High risk | Low risk | Low risk | High risk | High risk |
