# Supplementary material for: Interventions to prevent preterm birth following fetoscopic laser surgery for twin‐to‐twin transfusion syndrome: systematic review and meta‐analysis
Source: Ultrasound Obstet Gynecol. 2025 Jun 5;66(1):14–23. doi: 10.1002/uog.29230 (PMC12209700; doi:10.1002/uog.29230)
Supplement: Supplementary file 4 — Table S4 Pooled proportions (95% CI) for different outcomes explored in systematic review in monochorionic diamniotic twin pregnancies complicated by twin‐to‐twin transfusion syndrome undergoing fetoscopic laser surgery, according to type of preterm birth prevention strategy [file UOG-66-14-s002.docx]

**Table S4** Pooled proportions (95% CI) for different outcomes explored in systematic review in monochorionic diamniotic twin pregnancies complicated by twin-to-twin transfusion syndrome undergoing fetoscopic laser surgery, according to type of preterm birth prevention strategy

| **Outcome** | **Studies (n)** | **Cases (n/N)** | **Pooled proportions**  **(95% CI)** | **I^2^ (%)** |  | **Studies (n)** | **Cases (n/N)** | **Pooled proportions**  **(95% CI)** | **I^2^ (%)** |  |  |  |  |  |
| --- | --- | --- | --- | --- | --- | --- | --- | --- | --- | --- | --- | --- | --- | --- |
| ***Pregnancies undergoing intervention*** | | | | |  | ***Pregnancies NOT undergoing intervention*** | | | |  |  |  |  |  |
| ***Cervical cerclage*** | | | | | | | | | |  |  |  |  |  |
| ***Cervical cerclage in women with CL <30 mm*** | | | | | | | | | |  |  |  |  |  |
| Preterm birth < 34 weeks | 2 | 18/23 | 76.57 (57.97-90.96) | 0 |  | - | - | - | - |  |  |  |  |  |
| Preterm birth < 32 weeks | 5 | 58/75 | 76.18 (66.13-84.94) | 0 |  | 2 | 116/212 | 50.21 (27.69-72.68) | 89.9 |  |  |  |  |  |
| Preterm birth < 28 weeks | 5 | 38/75 | 50.23 (35.01-65.39) | 45.8 |  | 2 | 62/212 | 28.68 (20.90-37.15) | 0 |  |  |  |  |  |
| Preterm birth < 24 weeks | 4 | 15/65 | 21.46 (5.02-45.20) | 77.1 |  | 2 | 26/212 | 11.34 (4.98-19.87) | 61.9 |  |  |  |  |  |
| Delivery within 4 weeks from laser | 3 | 18/56 | 29.55 (9.16-55.66) | 76.2 |  | 2 | 54/212 | 25.70 (20.07-31.79) | 0 |  |  |  |  |  |
| Delivery within 2 weeks from laser | 3 | 13/56 | 21.97 (6.10-44.10) | 70.1 |  | 2 | 36/212 | 17.27 (12.50-22.63) | 0 |  |  |  |  |  |
| PPROM | 4 | 37/119 | 31.73 (15.21-51.06) | 69.8 |  | 2 | 45/142 | 48.50 (9.68-88.47) | 65.5 |  |  |  |  |  |
| Chorioamnionitis | 3 | 7/167 | 4.89 (1.06-11.32) | 50 |  | 2 | 4/89 | 5.22 (1.61-10.73) | 0 |  |  |  |  |  |
| Double survival (fetus) | 2 | 67/97 | 64.96 (43.92-83.33) | 69.4 |  | 1 | 42/61 | 68.85 (55.71-80.10) | - |  |  |  |  |  |
| At least on survivor (fetus) | 2 | 82/97 | 79.79 (54.15-96.48) | 81.6 |  | 1 | 52/61 | 85.25 (73.83-93.02) | - |  |  |  |  |  |
| No survivor (newborn) | 2 | 15/97 | 20.21 (35.18-45.85) | 81.6 |  | 1 | 9/61 | 14.75 (6.98-26.17) | - |  |  |  |  |  |
| Overall fetal or perinatal loss | 3 | 37/112 | 30.55 (13.81-50.51) | 79.5 |  | 2 | 124/396 | 29.34 (18.41-41.63) | 83 |  |  |  |  |  |
| Overall fetal or perinatal survival | 3 | 75/112 | 69.45 (49.49-86.19) | 79.5 |  | 2 | 272/396 | 70.66 (58.37-81.59) | 83 |  |  |  |  |  |
|  | | | | | | | | | |  |  |  |  |  |
| ***Cervical cerclage in women with CL <25 mm*** | | | | | | | | | |  |  |  |  |  |
| Preterm birth < 34 weeks | 2 | 18/23 | 76.57 (57.97-90.96) | 0 |  | - | - | - | - |  |  |  |  |  |
| Preterm birth < 32 weeks | 4 | 40/54 | 72.82 (60.52-83.56) | 0 |  | 1 | 23/61 | 37.70 (26.61-50.25) | - |  |  |  |  |  |
| Preterm birth < 28 weeks | 4 | 26/54 | 48.12 (28.48-68.07) | 56.6 |  | 1 | 14/61 | 22.95 (14.19-34.91) | - |  |  |  |  |  |
| Preterm birth < 24 weeks | 3 | 12/44 | 23.76 (1.81-59.74) | 84 |  | 1 | 4/61 | 6.56 (2.58-15.69) | - |  |  |  |  |  |
| Delivery within 4 weeks from laser | 2 | 12/35 | 28.99 (0.7-75.18) | 88 |  | 1 | 15/61 | 24.59 (15.51-36.68) | - |  |  |  |  |  |
| Delivery within 2 weeks from laser | 2 | 10/35 | 25.05 (1.76-62.86) | 81.9 |  | 1 | 9/61 | 14.75 (7.96-25.72) | - |  |  |  |  |  |
| PPROM | 3 | 32/102 | 26.74 (11.06-46.26) | 61.2 |  | 2 | 27/89 | 47.52 (7.57-89.61) | 80.6 |  |  |  |  |  |
| Chorioamnionitis | 2 | 2/88 | 4.72 (0.35-19.33) | 55.5 |  | 2 | 4/89 | 5.22 (1.61-10.73) | 0 |  |  |  |  |  |
| Double survival (fetus) | 2 | 66/10 | 63.33 (46.88-78.32) | 52.4 |  | 2 | 90/145 | 62.45 (50.93-73.29) | 50.6 |  |  |  |  |  |
| At least on survivor (fetus) | 2 | 84/100 | 79.34 (54.90-95.76) | 80 |  | 2 | 115/145 | 79.57 (68.95-88.44) | 55 |  |  |  |  |  |
| No survivor (fetus) | 2 | 16/100 | 20.66 (4.24-45.10) | 80 |  | 2 | 30/145 | 20.43 (11.56-31.05) | 55 |  |  |  |  |  |
| Double survival (newborn) | 2 | 66/100 | 63.33 (46.88-78.32) | 52.4 |  | 2 | 90/145 | 62.45 (50.93-73.29) | 50.6 |  |  |  |  |  |
| At least on survivor (newborn) | 2 | 84/100 | 79.34 (54.90-95.76) | 80 |  | 2 | 115/145 | 79.57 (68.95-88.44) | 55 |  |  |  |  |  |
| No survivor (newborn) | 2 | 16/100 | 20.66 (4.24-45.10) | 80 |  | 2 | 30/14552.4 | 20.43 (11.56-31.05) | 55 |  |  |  |  |  |
| Overall fetal or perinatal loss | 4 | 74/246 | 25.29 (13.62-39.13) | 74.7 |  | 3 | 91/300 | 32.72 (20.80-45.90) | 74.7 |  |  |  |  |  |
| Overall fetal or perinatal survival | 4 | 172/246 | 74.71 (60.87-86.38) | 74.7 |  | 3 | 209/300 | 67.28 (54.10-79.20) | 74.7 |  |  |  |  |  |
|  | | | | | | | | | |  |  |  |  |  |
| ***Cervical cerclage in women with CL <20 mm*** | | | | | | | | | |  |  |  |  |  |
| Preterm birth < 34 weeks | 2 | 18/23 | 76.57 (57.97-90.96) | 0 |  | - | - | - | - |  |  |  |  |  |
| Preterm birth < 32 weeks | 3 | 23/33 | 68.29 (52.04-82.53) | 0 |  | - | - | - | - |  |  |  |  |  |
| Preterm birth < 28 weeks | 3 | 14/33 | 44.75 (18.44-72.77) | 66 |  | - | - | - | - |  |  |  |  |  |
| Preterm birth < 24 weeks | 2 | 4/23 | 17.02 (2.76-72.39) | 88.1 |  | - | - | - | - |  |  |  |  |  |
| Delivery within 4 weeks from laser | - | - | - | - |  | - | - | - | - |  |  |  |  |  |
| Delivery within 2 weeks from laser | - | - | - | - |  | - | - | - | - |  |  |  |  |  |
| PPROM | - | - | - | - |  | - | - | - | - |  |  |  |  |  |
| Chorioamnionitis | 2 | 2/88 | 4.72 (0.35-19.33) | 55.5 |  | 2 | 4/89 | 5.22 (1.61-10.73) | 0 |  |  |  |  |  |
| Double survival (fetus) | - | - | - | - |  | - | - | - | - |  |  |  |  |  |
| At least on survivor (fetus) | - | - | - | - |  | - | - | - | - |  |  |  |  |  |
| No survivor (fetus) | - | - | - | - |  | - | - | - | - |  |  |  |  |  |
| Overall fetal or perinatal loss | 2 | 5/46 | 12.42 (4.64-23.24) | 0 |  | - | - | - | - |  |  |  |  |  |
| Overall fetal or perinatal survival | 2 | 21/46 | 87.58 (76.76-95.36) | 0 |  |  |  | - | - |  |  |  |  |  |
|  | | | | | | | | | |  |  |  |  |  |
| ***Cervical cerclage in women with CL <15 mm*** | | | | | | | | | |  |  |  |  |  |
| Preterm birth < 34 weeks | - | - | - | - |  | - | - | - | - |  |  |  |  |  |
| Preterm birth < 32 weeks | 2 | 16/24 | 65.56 (46.23-82.53) | 0 |  | - | - | - | - |  |  |  |  |  |
| Preterm birth < 28 weeks | 2 | 7/24 | 30.69 (14.54-48.78) | 0 |  | - | - | - | - |  |  |  |  |  |
| Preterm birth < 24 weeks | - | - | - | - |  | - | - | - | - |  |  |  |  |  |
| Delivery within 4 weeks from laser | - | - | - | - |  | - | - | - | - |  |  |  |  |  |
| Delivery within 2 weeks from laser | - | - | - | - |  | - | - | - | - |  |  |  |  |  |
| PPROM | 2 | 4/23 | 19.74 (2.33-48.21) | 56 |  | - | - | - | - |  |  |  |  |  |
| Chorioamnionitis | - | - | - | - |  | - | - | - | - |  |  |  |  |  |
| Double survival (fetus) | - | - | - | - |  | - | - | - | - |  |  |  |  |  |
| At least on survivor (fetus) | - | - | - | - |  | - | - | - | - |  |  |  |  |  |
| No survivor (fetus) | - | - | - | - |  | - | - | - | - |  |  |  |  |  |
| Overall fetal or perinatal loss | 2 | 5/46 | 12.42 (4.64-23.24) | 0 |  | - | - | - | - |  |  |  |  |  |
| Overall fetal or perinatal survival | 2 | 21/46 | 87.58 (76.76-95.36) | 0 |  | - | - | - | - |  |  |  |  |  |
|  | | | | | | | | | |  |  |  |  |  |
| ***Pessary in women with cervical short CL <30 mm*** | | | | | | | | | |  |  |  |  |  |
| Preterm birth < 34 weeks | - | - | - | - |  | - | - | - | - |  |  |  |  |  |
| Preterm birth < 32 weeks | 3 | 46/81 | 56.57 (45.80-67.03) | 9 |  | 3 | 119/220 | 48.04 (29.16-67.22) | 81.4 |  |  |  |  |  |
| Preterm birth < 28 weeks | 2 | 20/73 | 28.92 (16.10-43.76) | 38.8 |  | 2 | 62/212 | 28.67 (20.90-37.15) | 37.4 |  |  |  |  |  |
| Preterm birth < 24 weeks | 2 | 12/73 | 17.26 (9.57-26.64) | 0 |  | 2 | 26/212 | 11.34 (4.98-19.87) | 0 |  |  |  |  |  |
| Delivery within 4 weeks from laser | 2 | 17/73 | 27.60 (6.36-56.57) | 82.4 |  | 2 | 54/212 | 25.70 (20.07-31.78) | 0 |  |  |  |  |  |
| Delivery within 2 weeks from laser | 2 | 11/73 | 16.60 (6.73-29.74) | 40.3 |  | 2 | 36/212 | 17.27 (12.50-22.63) | 0 |  |  |  |  |  |
| PPROM | 4 | 13/89 | 15.77 (8.67-24.51) | 6.4 |  | 4 | 66/214 | 30.89 (23.87-38.38) | 12.8 |  |  |  |  |  |
| Chorioamnionitis | - | - | - | - |  | - | - | - | - |  |  |  |  |  |
| Double survival (fetus) | 3 | 58/80 | 82.35 (65.48-94.39) | 62.9 |  | 3 | 158/206 | 75.95 (67.49-83.48) | 30.8 |  |  |  |  |  |
| At least on survivor (fetus) | 3 | 75/80 | 86.76 (67.27-98.17) | 74.7 |  | 3 | 180/206 | 64.53 (27.52-93.52) | 95.3 |  |  |  |  |  |
| No survivor (fetus) | 3 | 7/80 | 12.29 (6.29-19.95) | 0 |  | 3 | 19/206 | 10.57 (4.88-18.10) | 43.7 |  |  |  |  |  |
| Overall fetal or perinatal loss | 3 | 46/160 | 34.20 (12.28-60.51) | 88.8 |  | 3 | 133/412 | 33.97 (21.95-47.14) | 79.8 |  |  |  |  |  |
| Overall fetal or perinatal survival | 3 | 114/160 | 65.80 (39.49-87.73) | 88.8 |  | 3 | 279/412 | 66.03 (52.86-78.05) | 79.8 |  |  |  |  |  |
| ***Pessary in women with cervical short CL <25 mm*** | | | | | | | | | |  |  |  |  |  |
| Preterm birth < 34 weeks | 2 | 33/60 | 54.85 (42.34-67.06) | 24.9 |  | 2 | 26/69 | 38.01 (27.07-49.61) | 0 |  |  |  |  |  |
| Preterm birth < 32 weeks | - | - | - | - |  | - | - | - | - |  |  |  |  |  |
| Preterm birth < 28 weeks | - | - | - | - |  | - | - | - | - |  |  |  |  |  |
| Preterm birth < 24 weeks | - | - | - | - |  | - | - | - | - |  |  |  |  |  |
| Delivery within 4 weeks from laser | - | - | - | - |  | - | - | - | - |  | - | - | - | - |
| Delivery within 2 weeks from laser | - | - | - | - |  | - | - | - | - |  |  |  |  |  |
| PPROM | 3 | 8/68 | 12.81 (6.05-21.61) | 0 |  | 3 | 25/77 | 27.94 (13.35-45.44) | 38.9 |  |  |  |  |  |
| Chorioamnionitis | - | - | - | - |  | - | - | - | - |  |  |  |  |  |
| Double survival (fetus) | 2 | 45/60 | 84.74 (49.53-99.98) | 78.8 |  | 2 | 49//69 | 70.56 (59.42-80.58) | 0 |  |  |  |  |  |
| At least on survivor (fetus) | 2 | 56/60 | 92.48 (84.60-97.70) | 0 |  | 2 | 52/69 | 75.02 (64.29-84.39) | 96.6 |  |  |  |  |  |
| No survivor (fetus) | 2 | 6/60 | 10.46 (3.87-19.76) | 2.9 |  | 2 | 10/69 | 15.43 (7.99-24.76) | 0 |  |  |  |  |  |
| Overall fetal or perinatal loss | 3 | 24/136 | 14.98 (2.97-33.89) | 76 |  | 3 | 39/154 | 29.08 (11.81-50.31) | 75 |  |  |  |  |  |
| Overall fetal or perinatal survival | 3 | 112/136 | 85.03 (66.11-97.03) | 76 |  | 3 | 115/154 | 70.92 (49.70-88.19) | 75 |  |  |  |  |  |

CL, cervical length; PPROM, preterm prelabor rupture of membranes.
