# Supplementary figures and images for: Interventions to prevent preterm birth following fetoscopic laser surgery for twin‐to‐twin transfusion syndrome: systematic review and meta‐analysis
Source: Ultrasound Obstet Gynecol. 2025 Jun 5;66(1):14–23. doi: 10.1002/uog.29230 (PMC12209700; doi:10.1002/uog.29230)

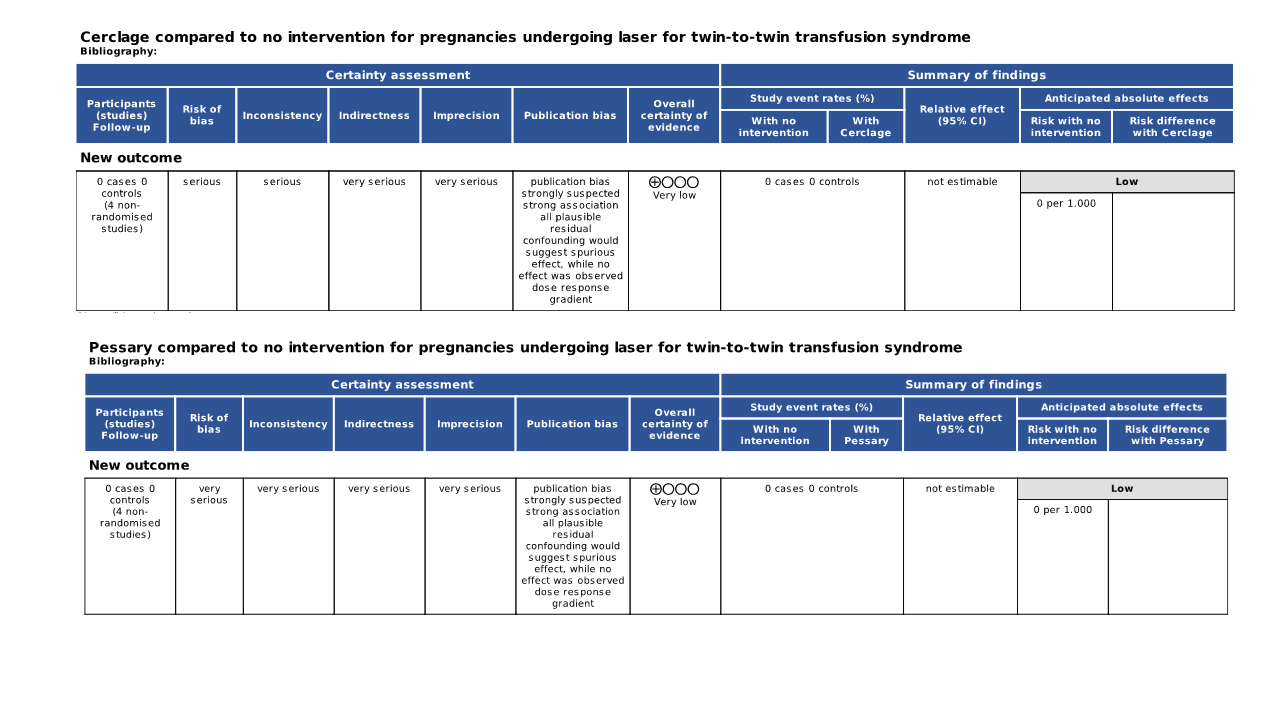

Supplement: Supplementary file 5 — Figure S1 Grading of Recommendations, Assessment, Development and Evaluation (GRADE) methodology for primary outcome. [file UOG-66-14-s003.tif]
